# METABOLOMICS OF DELIRIUM: A CASE-CONTROL STUDY

**DOI:** 10.1093/geroni/igz038.350

**Published:** 2019-11-08

**Authors:** Bridget Tripp, Simon Dillon, Sarinnapha Vasunilashorn, Long H Ngo, Tamara G Fong, Edward R Marcantonio, Towia A Libermann, Hasan Otu

**Affiliations:** 1 University of Nebraska-Lincoln, Lincoln, Nebraska, United States; 2 Beth Israel Deaconess Medical Center, Boston, Massachusetts, United States

## Abstract

Postoperative delirium complicates 15-50% of major surgery in older adults, resulting in poor patient outcomes and increased healthcare costs. Basic mechanisms of delirium are largely unknown. In this study we implemented a protocol that uses high-throughput metabolomics to explore potential biological mechanisms of delirium. We profiled the plasma metabolome of 10 delirium cases and 10 matched controls from the SAGES cohort of older adults without dementia undergoing major non-cardiac surgery. We used targeted mass spectrometry to measure 302 metabolites (features) preoperatively (PREOP) and on postoperative day 2 (POD2). Metabolomics studies are challenged by inherent technical variability and signal noise. With a small sample and a large feature count, signal noise diminishes statistical power and masks true biological signals. To address these challenges, we implemented quality control sample-based signal correction and normalization to internal standards (ISs). ISs were screened from a pool of 6 potential candidates, resulting in the removal of 3 that failed to perform well, while 3 were retained for our experiments. ISs also enabled successful concatenation of experiments run at different times. Prior to implementing quality control samples and customized ISs, no metabolites were identified as differentially expressed. After implementation, we identified one metabolite that was significantly differentially expressed at PREOP and 17 metabolites that were significantly differentially expressed at POD2 between delirium and controls (BH-corrected p-value < 0.05). In conclusion, integration of quality controls and normalization to internal standards enabled us to detect metabolites associated with postoperative delirium. Such methods should be considered for future metabolomics studies.

